# Glymphatic Dysfunction in Patients With Ischemic Stroke

**DOI:** 10.3389/fnagi.2021.756249

**Published:** 2021-11-08

**Authors:** Cheng Hong Toh, Tiing Yee Siow

**Affiliations:** ^1^Department of Medical Imaging and Intervention, Chang Gung Memorial Hospital at Linkou, Taoyuan, Taiwan; ^2^Chang Gung University College of Medicine, Taoyuan, Taiwan

**Keywords:** neuroimaging biomarkers, ischemic stroke, diffusion tensor imaging, ALPS (analysis along perivascular space) index, glymphatic function

## Abstract

**Objectives:** Rodent experiments have provided some insight into the changes of glymphatic function in ischemic stroke. The diffusion tensor image analysis along the perivascular space (DTI-ALPS) method offers an opportunity for the noninvasive investigation of the glymphatic system in patients with ischemic stroke. We aimed to investigate the changes of glymphatic function in ischemic stroke and the factors associated with the changes.

**Materials and Methods:** A total of 50 patients (mean age 56.7 years; 30 men) and 44 normal subjects (mean age 53.3 years; 23 men) who had preoperative diffusion-tensor imaging for calculation of the analysis along the perivascular space (ALPS) index were retrospectively included. Information collected from each patient included sex, age, time since stroke onset, infarct location, hemorrhagic change, infarct volume, infarct apparent diffusion coefficient (ADC), infarct fractional anisotropy (FA), and ALPS index of both hemispheres. Interhemispheric differences in ALPS index (infarct side vs. contralateral normal side) were assessed with a paired *t*-test in all patients. ALPS index was normalized by calculating ALPS ratios (right-to-left and left-to-right) for comparisons between patients and normal subjects. Comparisons of ALPS ratios between patients and normal subjects were performed using analysis of covariance with adjustments for age and sex. Linear regression analyses were performed to identify factors associated with the ALPS index.

**Results:** In patients, the mean ALPS index ipsilateral to infarct was 1.162 ± 0.126, significantly lower (*P* < 0.001) than that of the contralateral side (1.335 ± 0.160). The right-to-left ALPS index ratio of patients with right cerebral infarct was 0.84 ± 0.08, significantly lower (*P* < 0.001) than that of normal subjects (0.95 ± 0.07). The left-to-right ALPS ratio of patients with left cerebral infarct was 0.92 ± 0.09, significantly (*P* < 0.001) lower than that of normal subjects (1.05 ± 0.08). On multiple linear regression analysis, time since stroke onset (β = 0.794, *P* < 0.001) was the only factor associated with the ALPS index.

**Conclusion:** The ALPS index showed lower values in ischemic stroke suggesting impaired glymphatic function. Following initial impairment, the ALPS index increased with the time since stroke onset, which is suggestive of glymphatic function recovery.

## Introduction

The glymphatic system has been recently recognized as a pathway for waste clearance and maintaining fluid balance in the brain’s parenchymal interstitium (Rasmussen et al., 2018). Cerebrospinal fluid (CSF) from the subarachnoid space flows into brain parenchyma through periarterial spaces of the penetrating arteries and under the influence of Aquaporin-4 water channels mixes with parenchymal interstitial fluid. The interstitial fluid and its solutes then move into the perivenous and perineuronal spaces thereafter leaving the brain parenchyma. The discovery of the glymphatic system led to a new perspective on the pathogenesis of many brain diseases including ischemic stroke (Mestre et al., 2020).

Rodent experiments have provided some insights into the changes in glymphatic function associated with ischemic stroke. It is widely accepted that interstitial fluid clearance in the glymphatic system is reduced after ischemic infarct (Ji et al., 2021; Lv et al., 2021). Despite that substantial knowledge has been gained from animal studies, further research is necessary to confirm if findings regarding the glymphatic system of animals apply to humans. Understanding the role of the glymphatic system in the pathophysiological process of ischemic stroke may help develop treatments that promote poststroke functional recovery. Comprehensive human research on the glymphatic system, however, is limited by the invasiveness of current evaluation tools (e.g., intrathecal contrast medium injection) (Ringstad et al., 2018; Edeklev et al., 2019; Watts et al., 2019).

Diffusion magnetic resonance imaging (MRI) has emerged as a noninvasive tool for human glymphatic system assessment. Analysis along the perivascular space (ALPS) index is a diffusion metric derived from diffusion tensor imaging (DTI) (Taoka et al., 2017). It estimates the diffusivity along with the perivascular spaces of medullary veins and has been used to assess glymphatic activity in clinical conditions including Alzheimer’s disease (Taoka et al., 2017; Steward et al., 2021), normal pressure hydrocephalus (Yokota et al., 2019; Bae et al., 2021), Parkinson’s disease (Chen et al., 2021; McKnight et al., 2021), age-related iron deposition (Zhou W. et al., 2020), diabetic-associated cognitive impairment (Yang et al., 2020), and tumor-associated brain edema (Toh et al., 2021; Toh and Siow, 2021).

The ALPS index offers an opportunity for the noninvasive investigation of the human glymphatic system, and thus, we took advantage of this method to evaluate the glymphatic system in patients with ischemic stroke. We aimed to investigate the changes of the ALPS index, which suggests a glymphatic function in ischemic stroke, and factors associated with the changes.

## Materials and Methods

### Study Subjects

Approval for reviewing the clinical data and preoperative MRI studies of patients was obtained from our Institutional Review Board. Between 2014 and 2018, a total of 58 consecutive patients with a diagnosis of ischemic stroke underwent preoperative DTI at our institution. Stroke onset time for each patient in this study was the estimate entered in the clinical record by the stroke neurologist. Patients who had an unknown time of stroke onset were not included in the study.

A total of eight patients were excluded due to posterior circulation infarct (*n* = 1), previous nonlacunar infarct (*n* = 1), bilateral cerebral infarction (*n* = 1), previously skull or brain surgery (*n* = 3), and time since stroke onset >60 days (*n* = 2). Thus, a total of 50 patients were analyzed. No patients had begun any thrombolytic or other recanalization therapies at the time of their MRI studies.

Patients were divided into two groups based on infarct volume (≤20 cm^3^ vs. >20 cm^3^) (Meng and Ji, 2021) and time since stroke onset (≤14 days vs. >14 days) (Kanekar et al., 2012). According to a meta-analysis of research on predictive factors in ischemic stroke, the infarct volume cut-point of 50 ml is sensitive in differentiating favorable and unfavorable outcomes (Meng and Ji, 2021). On the other hand, restoration of blood–brain barrier, resolution of vasogenic edema, and cleaning up of necrotic tissue begin after 14 days (Kanekar et al., 2012).

As healthy controls, 44 subjects (21 women, 23 men; mean age 53.3 ± 9.9 years; age range 27–83 years) with normal brain MRI examinations were also included.

### Clinical and Imaging Information

Medical records and MRI studies of the patient were retrospectively reviewed to collect clinical and imaging information including sex, age, and time since stroke onset (time interval between MRI examination and stroke onset time), and infarct location (right or left cerebral hemisphere).

### Magnetic Resonance Imaging

All MRI studies were performed using a 3T unit (Magnetom Tim Trio, Siemens, Erlangen, Germany) with a 12-channel phased-array head coil. All examinations included T2-weighted, FLAIR, susceptibility-weighted, DTI, and T1-weighted sequences acquired in the transverse plane before and after administration of 0.1 mmol/kg body weight gadopentetate dimeglumine (Magnevist; Schering, Berlin, Germany).

Diffusion tensor imaging was performed using a single-shot EPI with the following parameters: TR ms/TE ms, 5,800/83; diffusion gradient encoding in 20 directions; *b* = 0, 1,000 s/mm^2^; FOV, 256 × 256 mm; matrix size, 128 × 128; section thickness, 2 mm; and number of signals acquired, 4. A total of 50–60 sections without intersection gaps were used to cover the cerebral hemispheres, brainstem, and cerebellum. Generalized autocalibrating partially parallel acquisitions (GRAPPA) with a reduction factor set at 2 were used during DTI acquisitions. Contrast-enhanced T1-weighted images (TR/TE, 2,000/2.63 ms; section thickness, 1 mm; TI, 900 ms; acquisition matrix, 224 × 256 and FOV, 224 × 256 mm) were acquired after completion of the DTI sequence.

### Image Post-processing and Analysis

A software nordicICE (nordic Image Control and Evaluation Version 2, Nordic Imaging Lab, Bergen, Norway) was used for all volume measurements and for processing of diffusion-tensor data. The diffusion-weighted images were co-registered to the non-diffusion-weighted (*b* = 0) images to minimize the artifacts induced by eddy current and subject motion. Fractional anisotropy (FA) and apparent diffusion coefficient (ADC) were calculated from diffusion-tensor data using standard algorithms (Mukherjee et al., 2008; Toh and Castillo, 2021).

All images were co-registered based on a 3D nonrigid transformation and mutual information (Sundar et al., 2007). The adequacy of registration was visually assessed, and manual adjustments were performed by changing transformation parameters of translation, rotation, and/or scaling as necessary. Two neuroradiologists (with 16 and 6 years of experience) independently performed all measurements.

### Measurement of Infarct Volume

**FIGURE 1:**
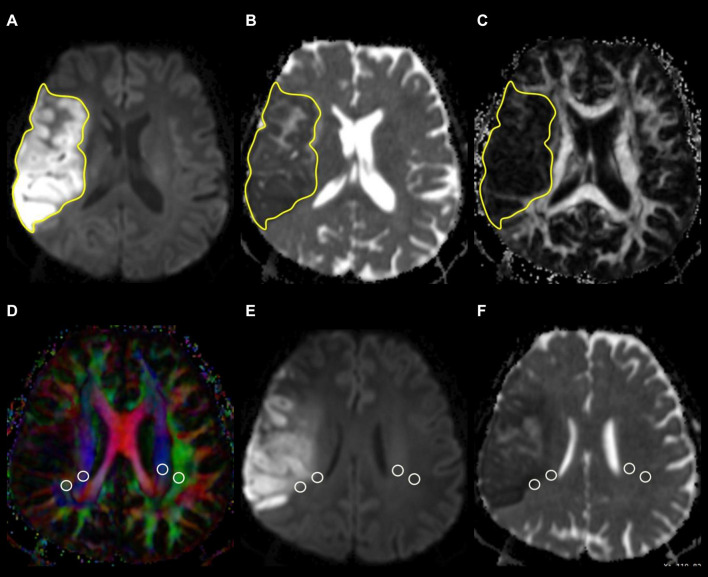
Example of how regions of interest (ROIs) were segmented. Transverse isotropic diffusion-weighted **(A)**, apparent diffusion coefficient **(B)**, and fractional anisotropy images show manually drawn ROIs for measurement of infarct volume, ADC, and FA values. Directionally encoded color map **(D)** illustrates ROIs of projection (blue area) and association (green area) fibers in bilateral periventricular regions. On the co-registered isotropic diffusion-weighted **(E)** and apparent diffusion coefficient **(F)** images, these ROIs do not include infarcted tissue.

The infarct volume was measured on FLAIR or isotropic DW images, depending on the lesion conspicuity and infarct stage, with reference to ADC, T2-weighted, and contrast-enhanced T1-weighted images. A polygonal region of interest (ROI) was manually drawn to include the entire infarct on each isotropic DW or FLAIR image. The ROIs on all images were then combined to form a whole infarct volume of interest for calculations of infarct volume. An example of ROI segmentation is shown in Figure 1.

### Measurements of Infarct Apparent Diffusion Coefficient and Fractional Anisotropy Values

Infarct ADC and FA values were measured using the same ROIs drawn on DW or FLAIR images. The ROIs were adjusted so as not to include regions with hemorrhagic change. Mean ADC and FA values of the whole infarct volume were calculated by averaging the values of all slices with the infarct volume of each slice taken into account.

### Measurement of Analysis Along the Perivascular Space Index

The DTI-ALPS method (Taoka et al., 2017, 2021) was used to evaluate the glymphatic function. This method evaluates the diffusivity along with the perivascular space on a transverse slice at the level of the lateral ventricle body. The medullary veins, accompanied by their perivascular spaces, run perpendicular to the ventricular walls at the level of the lateral ventricular bodies in a right-left or left-right direction (i.e., *x*-axis in image coordinate). In this level, the corticofugal corona radiata projection fibers run in the craniocaudal direction (i.e., *z*-axis in image coordinate) adjacent to the lateral ventricles. The superior longitudinal fascicle, which represents the association fibers, runs in the anterior-posterior direction (i.e., *y*-axis in image coordinate) and is located lateral to the corona radiata. As the perivascular space is nearly perpendicular to both the projection fibers and association fibers, the major difference between *x*-axis diffusivity in both fibers (*D*_xxpro__j_ and *D*_xxassoc_ for *x*-axis diffusivity in projection fiber and association fiber, respectively) and the diffusivity that is perpendicular to the *x*-axis and to the direction of fiber tracts (*y*-axis for projection fiber, where the diffusivity is denoted as *D*_yyproj_; *z*-axis for association fiber, where the diffusivity is denoted as *D*_zzassoc_) is the existence of perivascular space. To quantify glymphatic activity, the ALPS index is defined as follows:


(1) $$\mathrm{ALPS}\,\mathrm{index}=\frac{\text{mean}\,\left(D_{x\,x\,p\,r\,o\,j},D_{x\,x\,a\,s\,s\,o\,c}\right)}{\text{mean}\,\left(D_{y\,y\,p\,r\,o\,j},D_{z\,z\,a\,s\,s\,o\,c}\right)}$$

Diffusion metric images were generated by using 3D Slicer version 4.10.2.^1^ ROIs of projection (mean size, 18 ± 14 mm^2^) and association fibers (mean size, 16 ± 13 mm^2^) of both cerebral hemispheres were drawn on a slice at the level of the lateral ventricular body based on a directionality encoded map with reference to the co-registered ADC, isotropic DW, and FLAIR images so as not to include infarcted tissue. ALPS index was computed according to the Equation 1 above1. An example of ROI placement for ALPS index measurement is shown in Figure 1.

### Statistical Analysis

A commercially available statistical software package (SPSS 22; IBM, Armonk, NY, United States) was used for the analysis, and *P*-values < 0.05 were considered to indicate a statistical significance. Continuous variables are denoted as mean ± SD unless otherwise noted. The Kolmogorov–Smirnov test was used to assess the normality of continuous variables and guide the selection of a parametric or nonparametric test for the comparison of variables. Variance inflation factors were used to detect multicollinearity.

The interobserver variability in the measurements of infarct volume, ADC, FA, and ALPS index was assessed by intraclass correlation coefficients (ICCs) with 95% confidence intervals (CIs) based on an absolute-agreement, two-way random effects model. The final values of all measurements were obtained by taking the mean of the independent measurements of two observers.

To evaluate the changes of glymphatic function in ischemic strokes, interhemispheric differences in ALPS index (infarct side vs. contralateral normal side) were assessed with a paired *t*-test in all patients. ALPS index was normalized by calculating ALPS ratios (right-to-left and left-to-right) for comparisons between patients and normal subjects. For patients with right cerebral infarct, their right-to-left ALPS index ratio was compared with that of normal subjects. For patients with left cerebral infarct, their left-to-right ALPS index ratio was compared with that of normal subjects. The comparisons between patients and normal subjects were analyzed using analysis of covariance with adjustments for age and sex.

Among patients with ischemic stroke, group differences in ALPS index according to infarct volume (≤20 cm^3^ vs. >20 cm^3^) and time since stroke onset (≤14 days vs. >14 days) were assessed with analysis of covariance with adjustments for age and sex. Univariable linear regression analysis was first used to evaluate the associations of ALPS index with age, sex, infarct volume, infarct ADC, infarct FA, the presence of hemorrhagic change, and the time since stroke onset. All variables were entered as potential covariates in the stepwise multivariable linear regression analysis to identify independent factors associated with the ALPS index.

## Results

Among 50 patients (20 women, 30 men; mean age, 56.7 ± 15.2 years), 32 (64%) patients had right cerebral infarct and 19 (38%) patients showed hemorrhagic change. The mean time since stroke onset was 17.1 ± 14.8 days (range, 1–60). The mean infarct volume (cm^3^) of 50 patients was 34.07 ± 44.55 (range, 4.12–134.14). Table 1 summarizes patient characteristics and all measurements. There were excellent interobserver agreements in the measurements of infarct volumes (ICC = 0.802, 95% CI = 0.792–0.814, *P* < 0.001), infarct ADC (ICC = 0.956, 95% CI = 0.950–0.958, *P* < 0.001), infarct FA (ICC = 0.912, 95% CI = 0.906–0.918, *P* < 0.001), and bilateral ALPS indices (ICC = 0.838, 95% CI = 0.821–0.844, *P* < 0.001).

**TABLE 1:** Characteristics of patients and normal subjects.

Characteristics	Patient	Normal subject
No. of subjects	50	44
Mean age ± SD (year)	56.7 ± 15.2	53.3 ± 9.9
Sex		
Woman	20	21
Man	30	23
Infarct location		
Right cerebral	32	
Left cerebral	18	
Hemorrhagic change		
No	31	
Yes	19	
Infarct volume (cm^3^)	34.07 ± 44.55	
Infarct ADC (10^–6^ mm^2^/s)	709.13 ± 310.39	
Infarct FA	0.149 ± 0.086	
Time since stroke onset (day)	17.1 ± 14.8	
Mean ALPS index		
Ipsilateral to infarct	1.162 ± 0.126	
Contralateral to infarct	1.335 ± 0.160	

*Except where indicated, data are numbers of patients.*

*ADC, apparent diffusion coefficient; ALPS, analysis along the perivascular space;*

*FA, fractional anisotropy; SD, standard deviation.*

**FIGURE 2:**
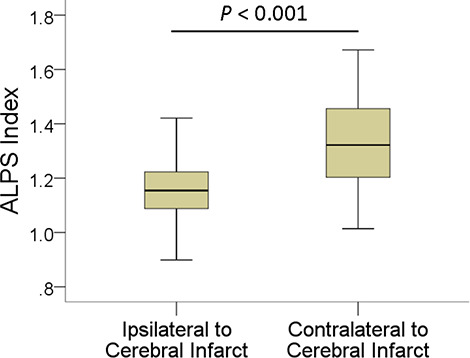
Boxplot shows interhemispheric differences in the ALPS index.

**FIGURE 3:**
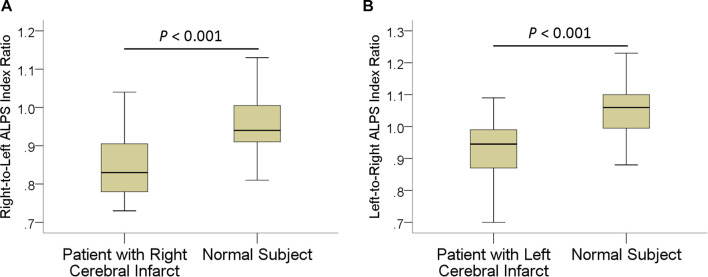
Boxplots show differences in right-to-left **(A)** and left-to-right **(B)** ALPS index ratios between patients with ischemic stroke and normal subjects.

In patients, the mean ALPS index ipsilateral to infarct was 1.162 ± 0.126, significantly (*P* < 0.001) lower than that of the contralateral side (1.335 ± 0.160). The interhemispheric differences in the ALPS index are illustrated in Figure 2. Comparisons of ALPS index ratios between patients and normal subjects are summarized in Table 2. The right-to-left ALPS index ratio of patients with right cerebral infarct was 0.84 ± 0.08, significantly (*P* < 0.001) lower than that of normal subjects (0.95 ± 0.07). The left-to-right ALPS ratio of patients with left cerebral infarct was 0.92 ± 0.09, significantly (*P* < 0.001) lower than that of normal subjects (1.05 ± 0.08). Figure 3 shows the differences in ALPS index ratios between patients and normal subjects.

**TABLE 2:** Comparisons of right-to-left and left-to-right ALPS index ratios between patients and normal subjects.

Variable	ALPS index ratio	*P-*value	95% CI
Right-to-left ALPS index ratio		<0.001	−13.99, −7.05
Patient with right cerebral infarct (*n* = 32)	0.84 ± 0.08		
Normal subject (*n* = 44)	0.95 ± 0.07		
Left-to-right ALPS index ratio		<0.001	−18.28, −7.79
Patient with left cerebral infarct (*n* = 18)	0.92 ± 0.09		
Normal subject (*n* = 44)	1.05 ± 0.08		

*Data are mean ± SD.*

*ALPS, analysis along the perivascular space; CI, confidence interval.*

**FIGURE 4:**
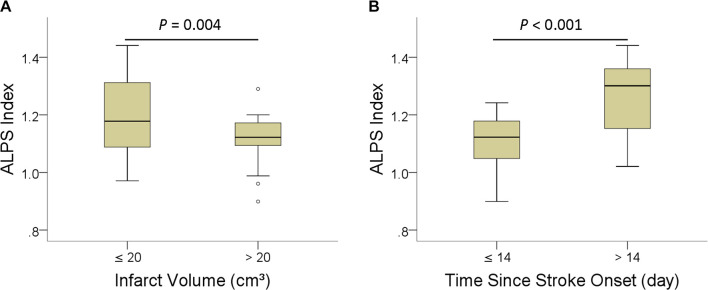
Boxplots of ALPS index with regard to infarct volume **(A)**, and **(B)** time since stroke onset.

Table 3 shows the group differences in ALPS index according to infarct volume (≤20 cm^3^ vs. >20 cm^3^) and time since stroke onset (≤14 days vs. >14 days). ALPS index was significantly higher in patients with infarct volume smaller than 20 cm^3^ (*P* = 004) and time since stroke onset longer than 14 days (*P* < 0.001). Figure 4 shows the differences in the ALPS index with regard to infarct volume and time since stroke onset.

**TABLE 3:** Group differences in ALPS index according to infarct volume and time since stroke onset.

Variable	ALPS index	*P-*value	95% CI
Infarct volume (cm^3^)		0.004	0.038, 0.181
≤20 (*n* = 33)	1.189 ± 0.133		
>20 (*n* = 17)	1.110 ± 0.094		
Time since stroke onset (day)		<0.001	0.093, 0.215
≤14 (*n* = 32)	1.108 ± 0.087		
>14 (*n* = 18)	1.259 ± 0.129		

*Data are mean ± SD.*

*ALPS, analysis along the perivascular space; CI, confidence interval.*

**FIGURE 5:**
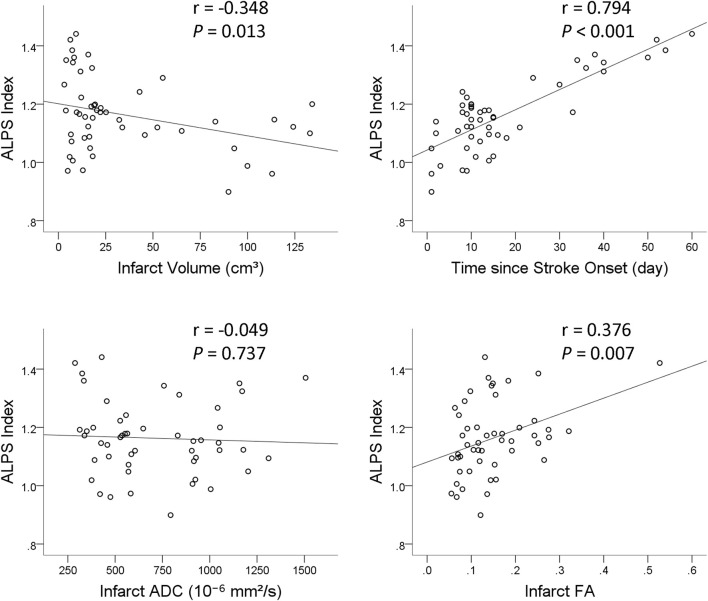
Scatterplots with regression line show the correlations of ALPS index with infarct volume, time since stroke onset, infarct apparent diffusion coefficient (ADC), and infarct fractional anisotropy (FA).

Results of univariable and multivariable linear regression analyses of factors associated with ALPS index are summarized in Table 4. On univariable linear regression analysis, ALPS index ipsilateral to the infarct correlated with infarct volume (β = −0.348, *P* = 0.013), infarct FA (β = 0.376, *P* = 0.007), presence of hemorrhagic change (β = −0.297, *P* = 0.036), and time since stroke onset (β = 0.794, *P* < 0.001). The associations of ALPS index with age (*P* = 0.594), sex (*P* = 0.061), and infarct ADC (*P* = 0.734) were not statistically significant. On stepwise multiple linear regression analysis, time since stroke onset (β = 0.794, *P* < 0.001) was the only factor associated with ALPS index. Figure 5 illustrates the correlations of ALPS index with infarct volume, infarct ADC, infarct FA, and time since stroke onset.

**TABLE 4:** Univariable and multivariable linear regression analyses of factors associated with ALPS index.

Characteristics	ALPS index							
	Univariate linear regressionh				Multivariate linear regression			
	B	SE	β	*P-*value	B	SE	β	*P-*value
Age	–0.001	0.001	–0.077	0.594				
Sex	–0.068	0.036	–0.267	0.061				
Infarct volume	–0.001	0.000	–0.348	0.013				
Infarct ADC	–0.002	0.000	–0.049	0.734				
Infarct FA	0.548	0.195	0.376	0.007				
Hemorrhagic change	–0.007	0.036	–0.297	0.036				
Time after stroke onset	0.007	0.001	0.794	<0.001	0.007	0.001	0.794	<0.001

*ALPS, analysis along the perivascular space; SD, standard deviation; B, unstandardized coefficient; β, standardized coefficient; SE, standard error.*

## Discussion

In this study, the glymphatic function was impaired in ischemic stroke, as reflected by lower ALPS index in cerebral hemispheres with infarct when compared with that of contralateral normal hemispheres and normal subjects. In addition, the ALPS index increased with the time since stroke onset, suggesting glymphatic function recovery following initial impairment. To our knowledge, this is the first human study reporting the dynamic changes of glymphatic function in ischemic stroke.

The ALPS index may serve as a marker of the function of interstitial fluid clearance as it measures the diffusivity and thus efflux rates of interstitial fluid in the perivascular spaces of deep medullary veins. The ALPS index has been shown to reflect glymphatic dysfunction in diseases known to have impaired clearance function. In patients with dementia, the ALPS index correlates with the Mini-Mental State Examination score of patients (Taoka et al., 2017). In patients with normal pressure hydrocephalus, there is a significant decrease in the ALPS index (Yokota et al., 2019; Bae et al., 2021). In this study, the ALPS index was significantly decreased in cerebral hemispheres with infarct and that may imply impaired glymphatic function and reduced interstitial fluid clearance, similar to that observed in animal studies (Ji et al., 2021; Lv et al., 2021).

It is widely accepted that interstitial fluid clearance is reduced after ischemic stroke in animal studies (Lv et al., 2021). Impaired CSF circulation in the glymphatic system after ischemic stroke has been a consistent finding (Gaberel et al., 2014; Yang et al., 2015; Lin et al., 2020). After ischemic stroke, there was a delay in the clearance of fluorescent traces in the infarct core (Zbesko et al., 2018) as well as amyloid deposits along with perivascular spaces (Arbel-Ornath et al., 2013). Using the middle cerebral artery occlusion model, a recent study found that glymphatic function in ipsilateral substantia nigra and ventral thalamic nucleus was impaired (Gaberel et al., 2014). In our study, we also observed a decrease of glymphatic function in the cerebral hemisphere with ischemic stroke, similar to what has been reported in animal studies. To the best of our knowledge, ischemic stroke-associated glymphatic function impairment has never been reported in human studies.

In this study, we observed a gradual increase of ALPS index with the time since stroke onset, as evidenced by an inverse association between the two. This finding may suggest glymphatic function recovery following initial impairment. The clinical significance of this phenomenon is unclear, and it may have important implications in the pathogenesis of poststroke dementia, which is one of the most common and severe consequences of stroke (Pantoni, 2017). It has been demonstrated that extracellular fluid present in infarct areas (i.e., liquefactive necrosis) is harmful to primary cultured cortical and hippocampal neurons (Zbesko et al., 2018). Impaired tau clearance, which has been implicated in the pathogenesis of poststroke dementia (Zhao et al., 2014), was observed in a study using a rat model of poststroke dementia (Back et al., 2020). In transgenic mice overexpressing human Slit2 (*Slit2-Tg*), cognition was improved *via* accelerating glymphatic clearance after ischemic stroke. Therefore, we speculate that the gradual increase of glymphatic function in patients with ischemic stroke serves to remove fluid and waste associated with tissue destruction in the ischemic infarct.

To date, no studies have reported the longitudinal or dynamic changes of glymphatic function in ischemic stroke. The mechanism of the glymphatic function recovery is not known, and we speculate that it may be the result of glymphatic pathway remodeling. As described in glioma-bearing mice, the glymphatic function is increased by extensive growth of meningeal lymphatic vessels, which is downstream of the glymphatic pathway (Zhou Y. et al., 2020; Wu et al., 2021). The glymphatic pathway remodeling helps fluid clearance and reduction of peritumoral brain edema (Hu et al., 2020). In mice with defective meningeal lymphatic vessels, impaired drainage of brain parenchymal interstitial fluid aggravates peritumoral brain edema. In meningiomas, remodeling of the glymphatic pathway has also been proposed to explain the inverse association between peritumoral edema volume and glymphatic function measured with the ALPS index (Toh et al., 2021). Further studies are needed to determine if glymphatic pathway remodeling occurs in ischemic infarct and its association with glymphatic function recovery.

The ADC and FA are useful in tissue characterization. ADC measures the magnitude of water diffusivity and reflects cytotoxic edema and changes of extracellular matrix volume in infarcted tissue (Nagaraja, 2021). ADC is useful in estimating the lesion age in ischemic stroke (Lansberg et al., 2001). FA, on the other hand, measures the directionality of water flow and may be associated with axonal injury after stroke (Bhagat et al., 2006). Although infarct volume (Vogt et al., 2012), infarct ADC (Nagaraja, 2021), infarct FA (Sotak, 2002; Moura et al., 2019), and hemorrhagic change (Kastrup et al., 2008) may predict outcomes of patients with ischemic stroke, they were not associated with the glymphatic function in our study. Rather, other factors such as Aquaporin-4 and meningeal lymphatics may have more significant roles in regulating the function of the glymphatic system (Lv et al., 2021).

Magnetic resonance imaging examinations performed after intrathecal injection of gadolinium-based contrast agents have confirmed the presence of the glymphatic system in the human brain (Zhou Y. et al., 2020). However, this method is invasive, and the off-label use of gadolinium-based contrast agents has potential neurotoxicity. DTI-ALPS method, in contrast, is noninvasive and has been shown to be highly reproducible in a recent study (Taoka et al., 2021). It allows investigating longitudinal changes of glymphatic function in the same subjects as well as comparison of glymphatic function across different patient groups. This method may help establish the predictive and prognostic roles of glymphatic function in patients with ischemic stroke.

In this study, the ischemic stroke-associated glymphatic dysfunction seen in animal studies was demonstrated in humans using the ALPS index. The ALPS index has been shown to reflect glymphatic function in several neurological disorders and to correlate with cognitive impairment (Taoka et al., 2017; Yang et al., 2020; Chen et al., 2021; Steward et al., 2021). However, its role as a neuroimaging biomarker of cognitive function in healthy elderly is not yet established. Recently, glymphatic failure has been proposed as a final common pathway to dementia (Nedergaard and Goldman, 2020). Hence, imaging markers that can detect glymphatic dysfunction and cognitive function decline may have important roles in early intervention. Our study further supports the ALPS index as a potential neuroimaging marker of glymphatic function and prospective investigation of the ALPS index as a measure of cognitive reserve in the normal aging process.

There are limitations to our study. First, the glymphatic system has only recently been recognized, and there are no well-established noninvasive methods to measure its function in humans. In our study, the efflux of interstitial fluid at perivenous space was measured. Further studies are needed to establish the correlation between ALPS index and interstitial fluid excretion function. Second, the imaging time points of patients in this study were between day 1 and day 60 after stroke onset. Longitudinal data on temporal changes of ALPS index in the individual patient was not available. These pieces of information would be helpful to confirm the phenomenon that glymphatic function recovers following initial impairment in patients with ischemic stroke. Third, the small sample size of our study may limit comprehensive subgroup analysis stratified by infarct volume and time since stroke onset as well as multivariate analysis of each potential factor associated with glymphatic function.

## Conclusion

In conclusion, the ALPS index showed lower values in ischemic stroke suggesting impaired glymphatic function. Following initial impairment, the ALPS index increased with the time since stroke onset, which is suggestive of glymphatic function recovery. However, larger series and prospective studies are needed to reach a definitive conclusion on changes in glymphatic function in patients with ischemic strokes.

## Data Availability Statement

The raw data supporting the conclusions of this article will be made available by the authors, without undue reservation.

## Ethics Statement

The studies involving human participants were reviewed and approved by the Chang Gung Medical Foundation Institutional Review Board. Written informed consent for participation was not required for this study in accordance with the national legislation and the institutional requirements.

## Author Contributions

CT and TS contributed to the conception and design of the study and the acquisition and analysis of the data. CT contributed to drafting the text and preparation of figures, and wrote the first draft of the manuscript. Both authors read and approved the final manuscript.

## Conflict of Interest

The authors declare that the research was conducted in the absence of any commercial or financial relationships that could be construed as a potential conflict of interest.

## Publisher’s Note

All claims expressed in this article are solely those of the authors and do not necessarily represent those of their affiliated organizations, or those of the publisher, the editors and the reviewers. Any product that may be evaluated in this article, or claim that may be made by its manufacturer, is not guaranteed or endorsed by the publisher.

## Footnotes

1
http://www.slicer.org
